# Ultrasound-triggered metabolic responses of *Lactobacillus acidophilus* at different growth phases enhance the functional quality of *Citrus aurantium* L. juice^☆^

**DOI:** 10.1016/j.ultsonch.2026.108120

**Published:** 2026-10-17

**Authors:** Binbin Li, Ningning Shen, Linxiao Wang, Shun Li, Longquan Xiao, Lin Zhou, Zhengnan Ren, Xinhui Wang

**Affiliations:** a College of Food and Biological Engineering, Chengdu University, Chengdu 610106, China; b Sichuan Provincial Engineering Research Center of Meat Quality Improvement and Safety Control Technology, Chengdu University, Chengdu 610106, China

**Keywords:** *Citrus aurantium* L., Ultrasound-assisted fermentation, Integrated untargeted metabolomics, Metabolic differences, Distinct metabolic routes

## Abstract

Accumulating evidence has demonstrated that ultrasound-assisted lactic acid bacteria (LAB) fermentation can improve the functional quality of fruit juices. However, the growth phase-dependent metabolic responses of LAB to low-frequency ultrasonication remain poorly understood. In this study, low-frequency ultrasonication (83.6 and 119.17 W/L) was applied during the lag, logarithmic, and stationary phases of *Lactobacillus acidophilus* LA137 fermentation in *Citrus aurantium* L. juice. Microbial growth, physicochemical properties, β-glucosidase activity, total phenolic and flavonoid contents, and antioxidant capacity were evaluated in this study. Ultrasonication applied during the logarithmic and stationary phases exerted greater effects on microbial metabolism and functional quality than treatment during the lag phase. Among all treatments, ultrasonication at 119.17 W/L during the logarithmic phase (LU4) and 83.6 W/L during the stationary phase (LU5) showed superior enhancement of β-glucosidase activity, phenolic/flavonoid accumulation, and antioxidant capacity. Integrated untargeted metabolomics revealed distinct metabolic responses between LU4 and LU5. LU4 preferentially promoted phenylpropanoid and flavonoid metabolism, resulting in the accumulation of antioxidant-related metabolites, including gallic acid, taxifolin, and 7-galloylcatechin. In contrast, LU5 mainly enhanced amino acid and organic acid metabolism, leading to increased levels of lactic acid, γ-aminobutyric acid, alanine, and serine. Correlation analysis further linked these metabolites to improved antioxidant properties. These findings demonstrate that low-frequency ultrasonication redirects microbial metabolism toward distinct functional routes depending on the growth phase of *L. acidophilus*, providing new mechanistic insights into the precise regulation of ultrasound-assisted LAB fermentation.

## Highlights

• Growth phase determined metabolic responses to low-intensity ultrasonication.
• Log-phase sonication enhanced antioxidant-related metabolite accumulation.
• Stationary-phase sonication favored amino acid and organic acid metabolism.
• Log- and stationary- phase sonication generated distinct metabolic signatures.
• Metabolomics revealed growth phase-dependent regulation of LAB fermentation.

## Graphical abstract

**Figure:**
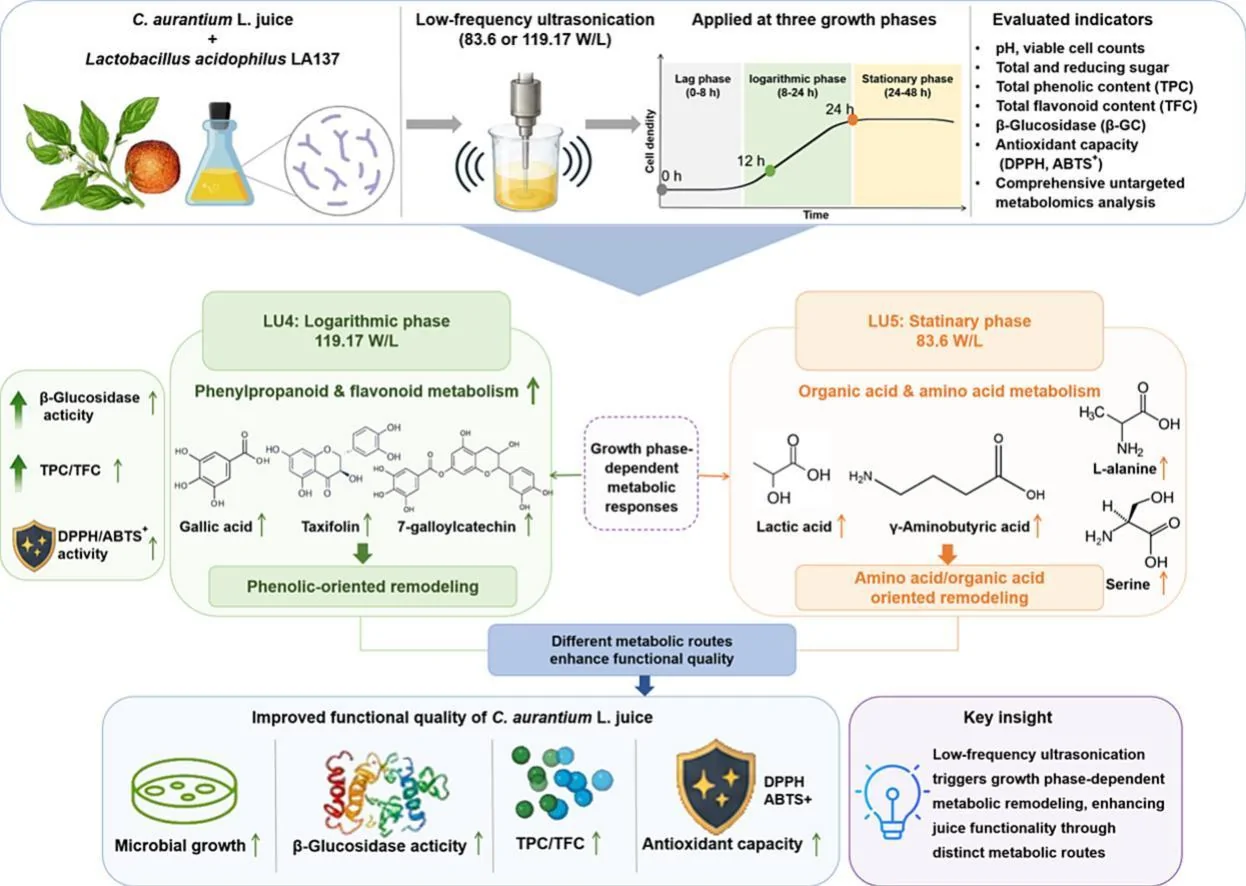

## 1 Introduction

*Citrus aurantium* L. (*C. aurantium*), commonly known as bitter orange, is a citrus fruit widely cultivated in tropical and subtropical regions and has attracted increasing attention owing to its nutritional value and health-promoting properties. *C. aurantium* is rich in flavonoids, phenolic acids, alkaloids, organic acids, dietary fibers, vitamins, and essential oils, which contribute to its characteristic flavor and diverse biological activities [1]. Previous studies have demonstrated that these bioactive compounds possess antioxidant, anti-inflammatory, antimicrobial, anti-obesity, and neuroprotective properties, highlighting their potential in the prevention and management of various chronic diseases [2], [3]. Despite these advantages, the effective utilization of *C. aurantium* remains challenging due to its highly acidic environment and abundant dietary fiber content, which may limit nutrient accessibility and the release of bound bioactive compounds. These characteristics reduce the efficient utilization of substrates and constrain the development of value-added products. Therefore, effective bioprocessing strategies are required to improve the conversion of endogenous bioactive compounds and maximize the functional potential of *C. aurantium*.

Lactic acid bacteria (LAB) fermentation is widely recognized as an effective bioprocessing strategy for improving the nutritional, sensory, and functional properties of fruit and vegetable products [4], [5]. Through microbial metabolism, LAB can release bound phenolics, transform flavonoid glycosides into bioactive derivatives, generate desirable flavor compounds, and enhance antioxidant capacity [6]. In addition, LAB fermentation can reduce anti-nutritional factors, improve microbial safety, and contribute to the development of novel functional products. Recently, low-frequency ultrasonication has emerged as a promising non-thermal technology for improving fermentation performance. Previous studies have demonstrated that ultrasonication can promote microbial growth, accelerate substrate utilization, and enhance metabolite production by improving membrane permeability, nutrient transport, and mass transfer efficiency [7], [8]. However, accumulating evidence suggests that the biological effects of ultrasonication are not solely determined by ultrasonic parameters. Instead, microbial responses to ultrasound are highly dependent on the physiological state of cells. Because microbial metabolism undergoes substantial changes throughout different growth phases, cells at distinct physiological stages may exhibit different sensitivities and metabolic responses to the same ultrasonic stimulus [9], [10]. Consequently, ultrasonication applied during different growth phases may redirect metabolic fluxes toward different biochemical pathways, ultimately leading to distinct fermentation outcomes and product qualities. Nevertheless, the mechanisms underlying these growth phase-dependent responses remain poorly understood.

*Lactobacillus acidophilus* (*L. acidophilus*) is a well-established probiotic species widely used in fruit juice fermentation owing to its excellent acid tolerance, probiotic functionality, and metabolic capacity for converting complex substrates into beneficial metabolites [11], [12]. Recent studies have reported that ultrasonication applied at different growth stages can induce distinct physiological responses, including variations in microbial proliferation, membrane permeability, stress adaptation, and metabolite biosynthesis [9], [10]. However, existing studies have primarily focused on the overall enhancement of fermentation performance by ultrasonication, while little attention has been paid to how the physiological state of microorganisms governs their metabolic responses to ultrasonic stimulation. In particular, it remains unclear whether ultrasonication applied during the logarithmic and stationary phases drives different metabolic routes and consequently leads to distinct functional attributes of fermented products. Moreover, previous investigations have mainly focused on fermentation performance indicators, whereas the underlying metabolic mechanisms involving both volatile and non-volatile metabolites have rarely been systematically explored. Therefore, integrated metabolomics provides a powerful approach for elucidating growth phase-dependent metabolic responses and uncovering the biochemical basis underlying ultrasonic regulation of fermentation processes.

Therefore, this study investigated the effects of low-frequency ultrasonication applied at different growth stages of *L. acidophilus* fermentation on the physicochemical characteristics and functional quality of *C. aurantium* juice. More importantly, integrated untargeted metabolomics covering both volatile and non-volatile metabolites was employed to elucidate the growth phase-dependent metabolic responses induced by ultrasonication. Particular emphasis was placed on comparing logarithmic-phase and stationary-phase ultrasonication to determine whether different physiological states of *L. acidophilus* redirect metabolism toward distinct functional routes, thereby generating different metabolic signatures and quality attributes. This work provides new mechanistic insights into the interaction between ultrasonication and microbial physiology and contributes to the precise regulation of ultrasound-assisted fermentation processes.

## 2 Materials and methods

### 2.1 Materials

*C. aurantium* fruits free of visible mold and disease were purchased from a local supermarket in Chengdu, China. The assay kits used in this study, including the reducing sugar and total sugar content assay kits, total polyphenol and total flavonoid content assay kits, 2,2-diphenyl-1-picrylhydrazyl (DPPH) and 2,2′-azino-bis (3-ethylbenzothiazoline-6-sulfonic acid) diammonium salt (ABTS^+^) radical scavenging capacity assay kits, and β-glucosidase (β-GC) activity assay kit were obtained from Solarbio Biotechnology Co., Ltd. (Beijing, China). *L. acidophilus* LA137 is a laboratory-preserved strain previously isolated by our team. *L. acidophilus* LA137 was cultured overnight on the De Man, Rogosa and Sharpe (MRS) agar (Hopebio Technology Co., Ltd., Qingdao, China) at 37 °C. A single colony was subsequently inoculated into MRS broth and incubated at 37 °C.

### 2.2 Growth curve determination of *L. acidophilus*

2 % of *L. acidophilus* LA137 culture was inoculated into MRS broth and the OD600 nm was monitored from 0 h to 48 h to evaluate the growth of *L. acidophilus.*

### 2.3 Ultrasound-assisted fermentation of *C. aurantium* juice

After washing with deionized water, the *C. aurantium* fruits were juiced with deionized water at a fruit-to-water ratio of 1:2 (w/v). The resulting juice was filtered through sterile gauze, and its pH was adjusted to 4.5. Juice receiving neither bacterial inoculation nor ultrasound treatment was designated as the untreated control group (C). For all fermentation treatments, the prepared juice was inoculated with 3 % (v/v) *L. acidophilus* LA137 to achieve an initial viable cell count of approximately 1 × 10^7^ colony-forming unit (CFU)/mL in the juice immediately after inoculation. The inoculated juice without ultrasound treatment was designated as the fermentation control group (L). All inoculated samples were incubated at 37 °C for 48 h. Ultrasound treatments were performed using a probe-type ultrasonic processor (SCIENTZ-IID, Ningbo Scientz Biotechnology Co., Ltd., Zhejiang, China) operating at a frequency of 20 kHz and equipped with a 6-mm-diameter probe. The working volume was 100 mL. The instrument-displayed nominal ultrasound powers were 8.36 and 11.917 W, corresponding to nominal volumetric power densities of 83.6 and 119.17 W/L, based on the working volume, respectively [14].

Samples were treated in pulsed mode (5 s on/5 s off) for a total elapsed processing time of 10 min. Accordingly, the effective ultrasound-on time (*t*) was 300 s. The energetic parameters were determined and calculated following the calorimetric approach described by Moura et al. [15]. Acoustic power (AP), acoustic intensity (AI), power density (PD), specific energy (SE), and energy density (ED) were calculated according to Equations (1)-(5):

(1) $$\mathrm{AP}\left(W\right)=mCp\left(\frac{\mathrm{dT}}{\mathrm{dt}}\right)$$

(2) $$\mathrm{AI}\left(\frac{W}{\mathrm{cm}{\;}^{2}}\right)=\frac{4\times AP}{\pi D^{2}}$$

(3) $$\mathrm{PD}\left(\frac{W}{\mathrm{mL}}\right)=\frac{\mathrm{AP}}{V}$$

(4) $$\mathrm{SE}\left(\frac{J}{g}\right)=\frac{\mathrm{AP}\times t}{m}$$

(5) $$\mathrm{ED}\left(\frac{J}{{\mathrm{cm}}^{3}}\right)=\frac{\mathrm{UP}\times t}{V}$$

where *m* is the sample mass (approximately 100 g); *Cp* is the specific heat capacity of the juice; *dT/dt* is the heating rate concerning process time (℃/s); *D* is the probe-tip diameter (0.6 cm); *V* is the working volume (100 mL); *UP* is the instrument-displayed nominal ultrasound power, and *t* is the effective ultrasound-on time. A specific heat capacity (*Cp*) of 3.73 J g^−1^℃^-1^, as previously reported for orange juice, was adopted for the present citrus juice system [16]. The measured initial heating rates were 0.02 and 0.03 °C/s for the nominal volumetric power densities of 83.6 and 119.17 W/L, respectively. For the treatment with a nominal input power density of 83.6 W/L, the calculated AP, AI, PD, SE, and ED values were 7.46 W, 26.38 W/cm^2^, 0.0746 W/mL (74.60 W/L), 22.38 J/g, and 25.08 J/cm^3^, respectively. The corresponding values for the treatment with a nominal input power density of 119.17 W/L were 11.19 W, 39.58 W/cm^2^, 0.1119 W/mL (111.90 W/L), 33.57 J/g, and 35.75 J/cm^3^, respectively.

The ultrasound-treated groups were designated according to the treatment time and nominal volumetric power density: LU1 and LU2 received 83.6 and 119.17 W/L, respectively, at 0 h of fermentation; LU3 and LU4 received 83.6 and 119.17 W/L, respectively, at 12 h; and LU5 and LU6 received 83.6 and 119.17 W/L, respectively, at 24 h.

### 2.4 Viable cell counts and pH assessment

The viable cell counts and pH values of juice were measured at 0, 6, 12, 24 and 36 h. Viable cell counts were determined by performing ten-fold serial dilutions of each sample in sterile saline, followed by plating on MRS agar. The plates were incubated at 37 °C for 72 h, and the results were expressed as Log CFU/mL. The pH values were recorded using a pH meter (Inesa Scientific Instrument Co., Ltd., Shanghai, China).

### 2.5 Reducing sugar and total sugar contents

Reducing sugar and total sugar contents were determined using commercial assay kits based on the 3,5-dinitrosalicylic acid (DNS) colorimetric method, with glucose as the calibration standard, according to the manufacturer’s instructions [17]. For total sugar determination, the samples were subjected to acid hydrolysis before reaction with DNS. Absorbance was measured at 540 nm. The calibration curve for total sugar was constructed using glucose standards ranging from 0.01 to 1.60 mg/mL and was described by the equation y = 0.8474x − 0.0393 (R^2^ = 0.9949). The calibration curve for reducing sugar was constructed using glucose standards ranging from 0.1 to 0.6 mg/mL and was described by the equation y = 6.1507x − 0.1795 (R^2^ = 0.9997). In both equations, x represents the glucose concentration (mg/mL), and y represents the absorbance at 540 nm.

### 2.6 Measurement of total phenolic content and total flavonoid content in fermented juice

Total phenolic content (TPC) was measured using a commercial assay kit according to the manufacturer’s instructions. A 0.1 mL aliquot of juice was extracted with 60 % ethanol under ultrasonic conditions (300 W, 60 °C, 30 min), followed by centrifugation at 12,000 rpm for 10 min. The absorbance was measured at 760 nm. The calibration curve was constructed using gallic acid standards (0.0049–0.625 mg/mL), yielding y = 3.4152x + 0.3024 (R^2^ = 0.9929). TPC was expressed as mg gallic acid equivalents per mL (mg GAE/mL). Total flavonoid content (TFC) was determined using a commercial assay kit, and the absorbance was measured at 470 nm. A rutin calibration curve (0.039–2.500 mg/mL) was used, yielding y = 0.905x − 0.0858 (R^2^ = 0.9985). TFC was expressed as mg rutin equivalents per mL (mg RE/mL). In both equations, x represents the standard concentration (mg/mL), and y represents the absorbance [18].

### 2.7 Assessment of β-glucosidase activity

β-Glucosidase (β-GC) activity was determined using a commercial enzyme activity assay kit as previously described [4].

### 2.8 Antioxidant properties of fermented juice

The antioxidant activities of the juice samples were assessed by measuring DPPH and ABTS^+^ radical scavenging capacities according to the manufacturer’s instructions [18].

### 2.9 Comprehensive untargeted metabolomics analysis

Comprehensive untargeted metabolomics analysis was performed using LC-MS and GC–MS platforms (Majorbio Biopharm Technology Co., Ltd., Shanghai, China). Untargeted LC-MS metabolomics analysis was performed using a UHPLC-Q Exactive HF system (Thermo Scientific, USA). Juice samples were extracted with methanol/acetonitrile (1:1, v/v) containing internal standards, followed by vortexing, ultrasonic extraction, centrifugation, nitrogen drying, and reconstitution. The obtained supernatants were subjected to LC-MS analysis. QC samples were prepared by pooling equal aliquots of all samples. Chromatographic separation was conducted using an ACQUITY UPLC HSS T3 column (100 mm × 2.1 mm, 1.8 μm; Waters, USA). Mass spectrometric data were acquired in both positive and negative ion modes. Raw data were processed using Progenesis QI v3.0 for peak extraction, alignment, and metabolite identification based on public and in-house databases. For GC–MS, the metabolomics analysis was carried out using an Orbitrap Exploris GC system (Thermo Scientific, Germany). Juice samples were extracted with methanol/acetonitrile (2:1, v/v) containing ribitol as the internal standard, followed by ultrasonic extraction, centrifugation, nitrogen drying, oximation, and BSTFA derivatization prior to GC–MS analysis. Metabolites were separated on a TG-5SILMS capillary column and detected in full-scan mode. The acquired data were processed using Compound Discoverer 3.3 software for peak extraction, alignment, and metabolite annotation based on the NIST 2023 database, GC-Orbitrap Metabolomics Library v2, and an in-house database.

### 2.10 Statistical analysis

All experiments were conducted in triplicate, and the results are expressed as mean ± standard deviation (SD). Statistical differences among groups were evaluated using one-way analysis of variance (ANOVA) followed by Tukey’s *post hoc* test. Data analysis was carried out using GraphPad Prism 10.0 (GraphPad Software, San Diego, CA, USA). Differences were considered statistically significant at *P* < 0.05.

## 3 Results and discussion

### 3.1 Effects of low-frequency ultrasonication applied at different growth phases on viable cell counts and pH

The growth curve of *L. acidophilus* was first determined to identify appropriate time points for ultrasonic intervention (Fig. 1). Based on the growth kinetics, the period from 0 to 8 h was assigned as the lag phase, 8–24 h as the logarithmic phase, and the period after 24 h as the stationary phase. Accordingly, low-frequency ultrasonication (83.6 and 119.17 W/L) was applied at 0 h, 12 h, and 24 h to investigate the influence of microbial physiological state on the response to ultrasonic stimulation.

**Fig. 1:**
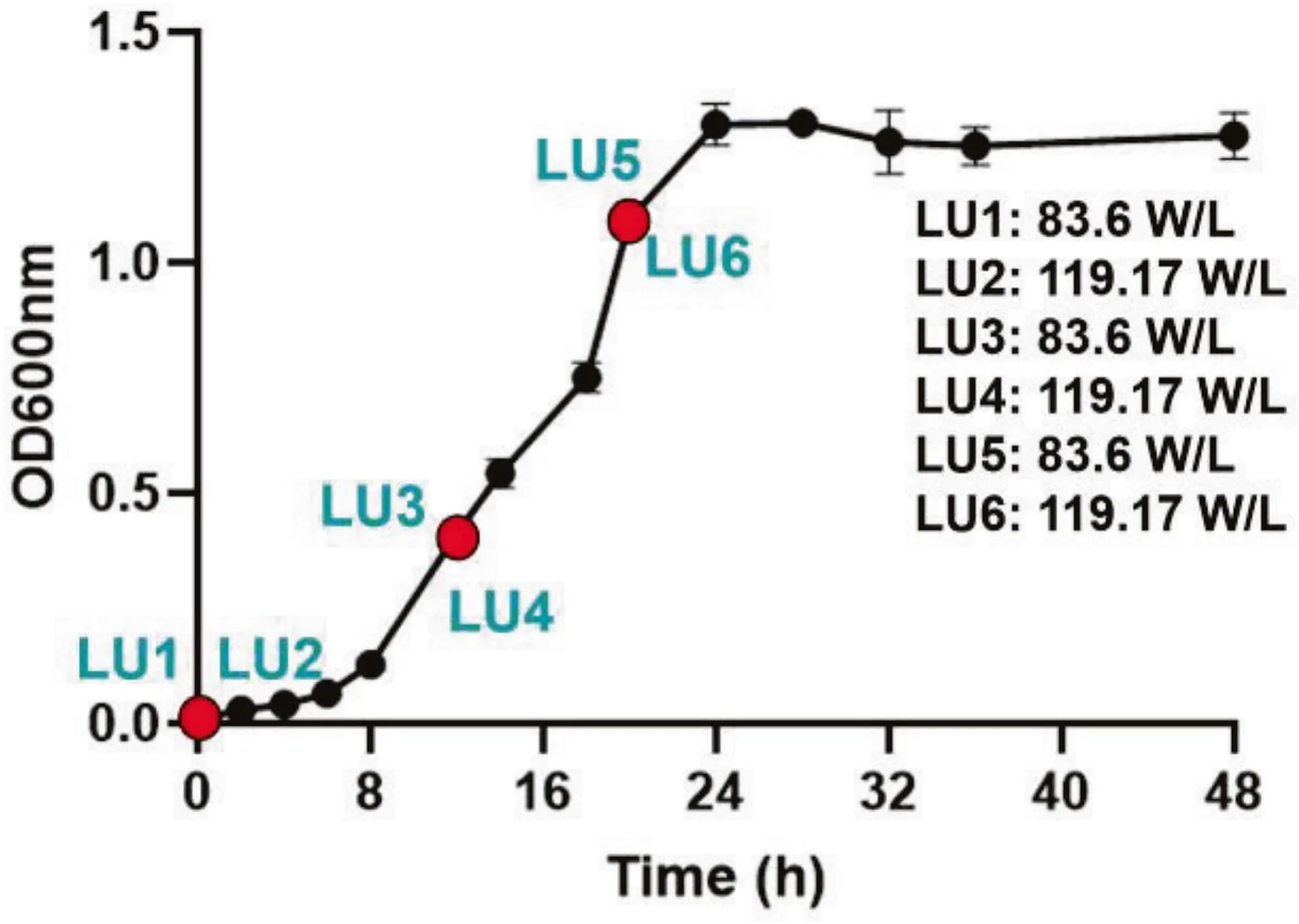
Growth curve of *Lactobacillus acidophilus* and experimental design of low-frequency ultrasonication applied at different growth phases. LU1 and LU2 were applied at the lag phase (0 h), LU3 and LU4 at the logarithmic phase (12 h), and LU5 and LU6 at the stationary phase (24 h), with nominal volumetric power densities of 83.6 and 119.17 W/L, respectively.

As shown in Fig. 2A, the viable cell counts of *L. acidophilus* increased rapidly during the first 24 h of fermentation and gradually declined thereafter. Ultrasonication generally promoted microbial proliferation, as all sonicated groups exhibited higher viable counts than the non-sonicated group. Among them, LU4 showed the highest cell density at 24 h (10.35 Log CFU/mL), followed by LU5 (10.1 Log CFU/mL) and LU3 (10.05 Log CFU/mL). Notably, ultrasonication applied during the logarithmic phase exerted a greater stimulatory effect on microbial growth than treatment during the lag phase. This observation suggests that the physiological state of *L. acidophilus* plays a critical role in determining its response to ultrasonic stimulation. Cells in the logarithmic phase are characterized by active metabolism, rapid nutrient uptake, and intensive biosynthesis, making them more responsive to ultrasound-induced enhancement of membrane permeability and mass transfer efficiency [19], [20]. Similar phase-dependent responses have been reported in other ultrasound-assisted LAB fermentation systems [13], [21].

**Fig. 2:**
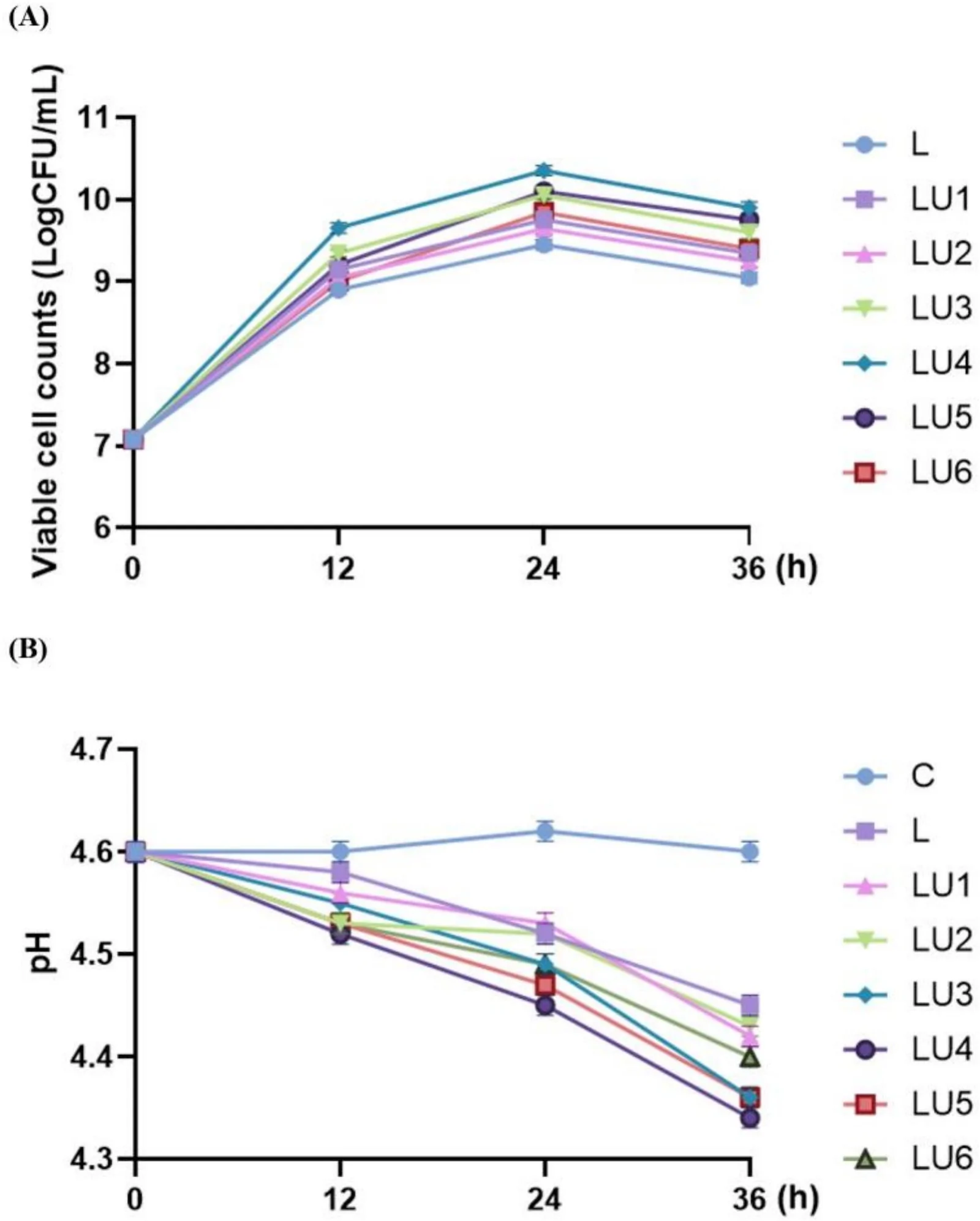
Effects of low-frequency ultrasonication applied at different growth phases on the viable cell counts and pH of *Lactobacillus acidophilus*-fermented *C. aurantium* L. juice. (A) Viable cell counts. (B) pH values. LU1 and LU2 were applied at the lag phase (0 h), LU3 and LU4 at the logarithmic phase (12 h), and LU5 and LU6 at the stationary phase (24 h), with ultrasonic intensities of 83.6 and 119.17 W/L, respectively.

The pH values of all fermented groups gradually decreased throughout fermentation (Fig. 2B), reflecting continuous carbohydrate metabolism and organic acid production. Compared with the non-sonicated group, ultrasonicated samples generally exhibited lower pH values, indicating enhanced metabolic activity [22], [23]. The lowest final pH was observed in LU4 (4.34 ± 0.01), followed by LU3 (4.36 ± 0.01) and LU5 (4.36 ± 0.01), which was consistent with their higher viable cell counts. Interestingly, although bacterial growth entered the stationary phase after 24 h, pH continued to decline, suggesting that metabolically active cells remained capable of converting residual carbohydrates into organic acids. This phenomenon indicates that microbial growth and metabolic activity were not completely synchronized during fermentation. Therefore, the superior acidification observed in LU5 may not solely result from increased cell numbers but also from enhanced metabolic activity induced by ultrasonication during the stationary phase. Similar observations have been reported in literature, where bacterial growth gradually slowed after entering the stationary phase, whereas pH continued to decrease due to ongoing carbohydrate utilization and organic acid accumulation [13]. In addition, metabolic intermediates such as pyruvate and malate can be further converted into organic acids, contributing to sustained acidification during the later stages of fermentation [9]. Collectively, these results demonstrate that low-frequency ultrasonication influences both microbial growth and metabolism in a growth phase-dependent manner. Logarithmic-phase ultrasonication primarily promoted cell proliferation, whereas stationary-phase ultrasonication appeared to sustain metabolic activity despite limited cell growth, implying that different physiological states may lead to distinct metabolic responses to ultrasonic stimulation.

### 3.2 Effects of low-frequency ultrasonication on total sugar and reducing sugar contents

Carbohydrate metabolism plays a central role in LAB fermentation by providing energy and metabolic precursors for microbial growth and metabolite biosynthesis [24], [25]. Therefore, variations in sugar utilization may reflect alterations in microbial metabolic activity induced by ultrasonication. As shown in Table 1, the total sugar contents of all fermented groups gradually decreased throughout fermentation, indicating continuous carbohydrate consumption by *L. acidophilus*.

**Table 1:** Total sugar content (mg/mL) of juice samples.

Fermentation time (h)	Group							
	C	L	LU1	LU2	LU3	LU4	LU5	LU6
0	43.34 ± 0.11^a^	43.4 ± 0.41^a^	42.2 ± 0.45^a^	43.22 ± 0.17^a^	42.85 ± 0.13^a^	43.04 ± 0.05^a^	42.9 ± 0.43^a^	42.66 ± 1^a^
12	43.11 ± 0.11^a^	41.63 ± 0.14^b^	41.1 ± 0.67^b^	42.27 ± 0.06^a^	41.6 ± 0.91^b^	41.71 ± 0.11^b^	40.79 ± 0.22^b^	41.31 ± 0.09^b^
24	42.24 ± 0.47^a^	41.27 ± 0.2^b^	40.77 ± 0.45^b^	40.68 ± 0.12^b^	40.56 ± 0.23^b^	39.06 ± 0.11^c^	38.39 ± 0.33^d^	39.72 ± 0.27^c^
36	41.34 ± 0.39^a^	40.33 ± 0.47^a^	39.47 ± 0.83^b^	38.23 ± 0.24^c^	37.59 ± 0.39^c^	37.53 ± 0.29^c^	37.22 ± 0.48^c^	37.97 ± 0.28^c^

Note: Data are presented as mean ± SD (n = 3). Different lowercase letters within the same row indicate significant differences among treatments at the same fermentation time according to Tukey’s multiple-comparison test (*P* < 0.05).

Compared with the unfermented control, fermentation markedly accelerated sugar depletion, whereas ultrasonication further enhanced this process. At 36 h, LU5 exhibited the lowest residual total sugar contents (37.22 ± 0.48 mg/mL) among all treatments, while LU3, LU4, and LU6 also exhibited relatively low levels. In contrast, LU1 and LU2 retained relatively higher sugar levels (39.47 ± 0.83 mg/mL and 38.23 ± 0.24 mg/mL), indicating a weaker stimulatory effect of this treatment condition on microbial metabolism. The decline in total sugar content reflects the utilization of fermentable carbohydrates as carbon and energy sources by *L. acidophilus*, thereby supporting microbial growth and metabolic activities [26].

The reducing sugar content showed a different trend, characterized by an initial increase during the early fermentation stage followed by a progressive decline (Table 2). The transient accumulation of reducing sugars at 12 h likely resulted from the hydrolysis of complex carbohydrates into readily fermentable monosaccharides, whereas the subsequent decrease reflected their rapid utilization by *L. acidophilus*. Notably, LU3 displayed the highest reducing sugar contents (23.78 ± 0.11 mg/mL) at 12 h. LU4 also showed a relatively high reducing sugar content (23.08 ± 0.06 mg/mL), although it did not differ significantly from the L and LU1 groups. These results suggest that ultrasonication during the logarithmic phase facilitated carbohydrate depolymerization and improved substrate availability. As fermentation proceeded, LU4 and LU6 showed the lowest reducing sugar levels (17.05 ± 0.03 mg/mL and 17.16 ± 0.11 mg/mL) at 36 h, indicating enhanced carbon utilization efficiency under these treatment conditions.

**Table 2:** Reducing sugar content (mg/mL) of juice samples.

Fermentation time (h)	Group							
	C	L	LU1	LU2	LU3	LU4	LU5	LU6
0	20 ± 0.06^a^	20.00 ± 0.03^a^	20.01 ± 0.06^a^	19.98 ± 0.04^a^	20.00 ± 0.08^a^	20.04 ± 0.04^a^	20.07 ± 0.05^a^	19.99 ± 0.02^a^
12	20.12 ± 0.07^d^	22.96 ± 0.05^b^	23.01 ± 0.04^b^	21.99 ± 0.02^c^	23.78 ± 0.11^a^	23.08 ± 0.06^b^	22.00 ± 0.02^c^	21.88 ± 0.05^c^
24	20.02 ± 0.01^b^	20.01 ± 0.1^b^	21.53 ± 0.59^a^	20.63 ± 1.14^ab^	21.64 ± 0.54^a^	19.96 ± 0.09^b^	20.05 ± 0.07^b^	20.01 ± 0.09^b^
36	19.87 ± 0.11^a^	18.97 ± 0.18^b^	19.05 ± 0.03^b^	18.81 ± 0.31^b^	18.91 ± 0.09^b^	17.05 ± 0.03^d^	17.84 ± 0.03^c^	17.16 ± 0.11^d^

Note: Data are presented as mean ± SD (n = 3). Different lowercase letters within the same row indicate significant differences among treatments at the same fermentation time according to Tukey’s multiple-comparison test (*P* < 0.05).

From a mechanistic perspective, the enhanced carbohydrate consumption observed in LU4 may be associated with the high physiological activity of cells during the logarithmic phase. Ultrasonication can increase membrane permeability and mass transfer efficiency, thereby facilitating nutrient uptake and accelerating carbohydrate catabolism [9], [13]. Although LU5 was treated during the stationary phase, it exhibited pronounced sugar depletion, indicating that ultrasonication sustained metabolic activity even after cell growth had plateaued. Combined with the viable cell count and pH results, these findings suggest that low-frequency ultrasonication influences carbohydrate metabolism in a growth phase-dependent manner. More importantly, the distinct patterns of sugar utilization observed in LU4 and LU5 imply that ultrasonic stimulation at different physiological stages may redistribute carbon flux toward different metabolic pathways, which could ultimately contribute to the distinct metabolic signatures.

### 3.3 Effects of low-frequency ultrasonication on total phenolic and flavonoid contents

Phenolic compounds, particularly flavonoids, are important bioactive constituents of *C. aurantium* and contribute substantially to its antioxidant potential and functional properties. As shown in Fig. 3A, fermentation significantly increased the TPC compared with the unfermented control, indicating that *L. acidophilus* promoted the release and transformation of phenolic compounds during fermentation. Ultrasonication further enhanced phenolic accumulation, although the extent of enhancement depended on both the growth phase and ultrasonic intensity. Among all treatments, LU3 exhibited the highest TPC (0.145 mg GAE/mL), representing a 22.9 % increase relative to the control group (0.118 mg GAE/mL), whereas LU4, LU5, and LU6 showed moderate but significant increases. The increase in TPC can be attributed to the synergistic effects of microbial fermentation and ultrasonication. During fermentation, microbial enzymes such as esterases and glycosidases facilitate the release of bound phenolics from the plant matrix, while ultrasonic cavitation enhances mass transfer and substrate accessibility, thereby promoting phenolic liberation [4]. Notably, the superior TPC observed in LU3 suggests that low-frequency ultrasonication applied during the logarithmic phase effectively stimulated the release of phenolic compounds. Cells at this stage are characterized by active metabolism and rapid nutrient uptake, making them more responsive to ultrasound-induced enhancement of membrane permeability and enzymatic activity in *L. acidophilus* [27]. Similar phenomena have been reported in ultrasound-assisted fermentations, where low intensity of ultrasound enhanced phenolic release, whereas high intensity of sonication promoted the conversion or degradation of phenolic substances [13]. These findings indicate that logarithmic-phase ultrasonication favored phenolic liberation and turnover during fermentation.

**Fig. 3:**
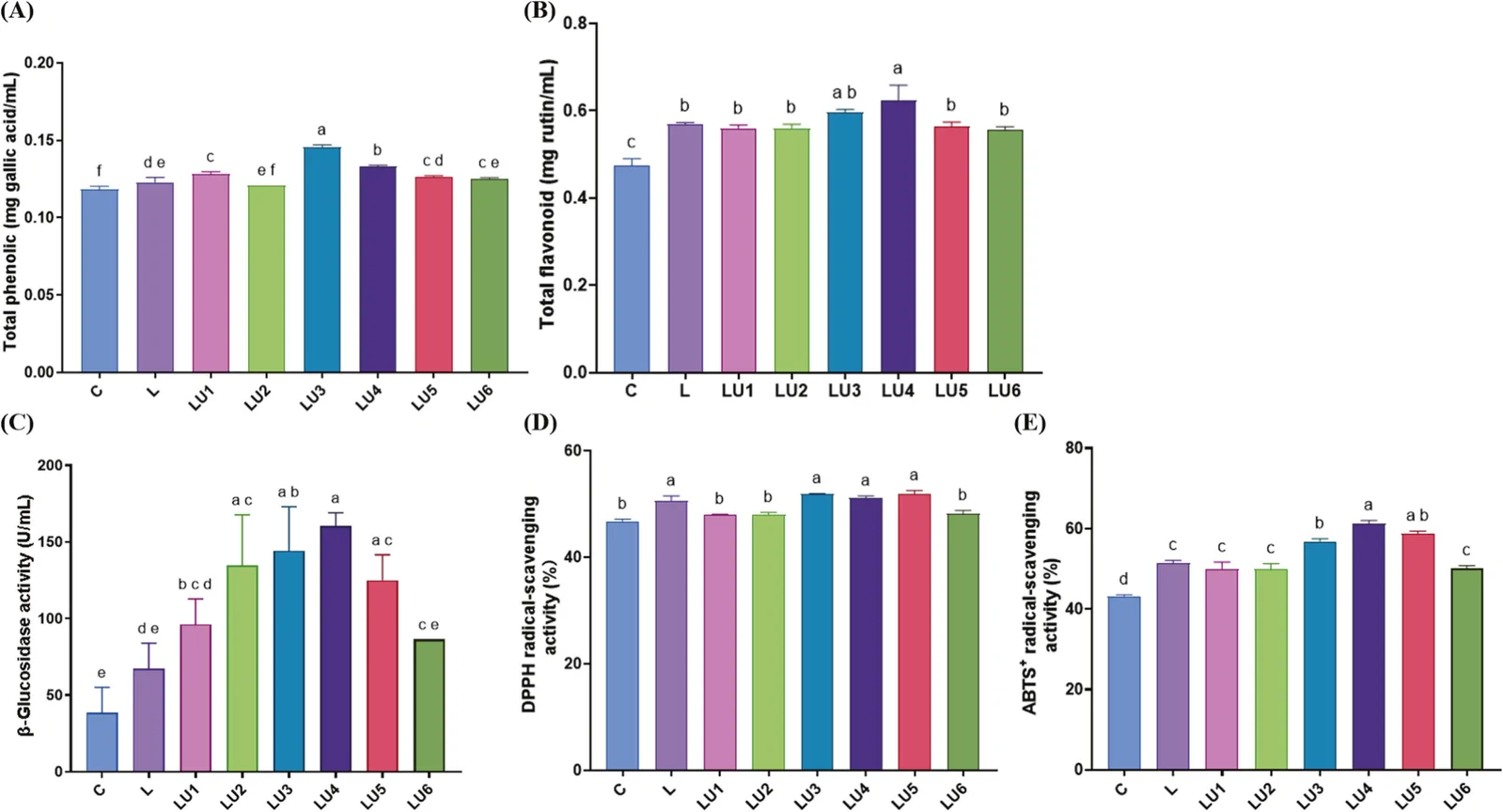
Growth phase-dependent effects of low-frequency ultrasonication on the functional quality and antioxidant properties of *L. acidophilus*-fermented *C. aurantium* L. juice. (A) Total phenolic content. (B) Total flavonoid content. (C) β-Glucosidase activity. (D) DPPH radical scavenging activity. (E) ABTS^+^ radical scavenging activity. LU1 and LU2 were applied at the lag phase (0 h), LU3 and LU4 at the logarithmic phase (12 h), and LU5 and LU6 at the stationary phase (24 h), with ultrasonic intensities of 83.6 and 119.17 W/L, respectively. Different letters indicate significant differences at the 0.05 level.

Flavonoids are important bioactive compounds that contribute substantially to the antioxidant activity and nutritional quality of fruit juices. As illustrated in Fig. 3B, the TFC was also significantly enhanced by fermentation and ultrasonication. Compared with the unfermented control (C, 0.47 mg RE/mL), fermentation increased TFC to 0.57 mg RE/mL, indicating that *L. acidophilus* fermentation promoted the release and transformation of flavonoid compounds. Ultrasound-assisted fermentation further enhanced TFC, with LU4 exhibiting the highest value (0.62 mg RE/mL), followed by LU3 (0.59 mg RE/mL). In contrast, LU5 and LU6 showed relatively lower TFC values, although they remained significantly higher than the control group. These findings indicate that ultrasonication applied during the logarithmic phase was more effective in enhancing flavonoid accumulation than that applied during the stationary phase. The observed enhancement of TFC following fermentation and ultrasound treatment may be associated with structural modifications and biotransformation of flavonoid compounds during processing. LAB fermentation is known to modify flavonoid composition through enzymatic reactions, particularly β-glucosidase-mediated deglycosylation and biotransformation of phenolic conjugates into more bioactive forms [28]. Such transformations can increase the extractability and measurable concentration of flavonoids in fruit-based substrates. In addition, ultrasonication can disrupt plant cellular structures and improve interactions between microbial enzymes and flavonoid substrates, thereby accelerating flavonoid conversion [29]. Similar enhancements in flavonoid accumulation following ultrasound-assisted fermentation have been reported by Yang et al. and Mamy et al. [10], [30].

### 3.4 Effects of low-frequency ultrasonication on β-glucosidase activity

β-GC is a key enzyme involved in the biotransformation of phenolic compounds during LAB fermentation. By hydrolyzing flavonoid glycosides into their corresponding aglycones, β-GC enhances the bioavailability and biological activity of phenolic compounds and contributes to the functional quality of fermented products [28], [31]. As shown in Fig. 3C, β-GC activity was significantly enhanced by both fermentation and ultrasonication. Compared with the unfermented control (38.24 U/mL), *L. acidophilus* fermentation increased β-GC activity to 66.87 U/mL, whereas ultrasound-assisted fermentation further stimulated enzyme production. Among all treatments, LU4 exhibited the highest β-GC activity (160.21 U/mL), followed by LU3, LU2 and LU5, indicating that ultrasonication applied during the logarithmic phase was most effective in promoting β-GC synthesis. In contrast, stationary-phase treatments showed a lower stimulatory effect, particularly LU6, which exhibited a marked decline in β-GC activity compared with LU4. These findings suggest that the enzymatic response to ultrasonic stimulation is strongly influenced by the physiological state of *L. acidophilus*. Cells in the logarithmic phase are characterized by active metabolism and intensive protein synthesis, making them more responsive to ultrasound-induced enhancement of membrane permeability, nutrient transport, and metabolic activity [10], [13].

The elevated β-GC activity observed in LU3 and LU4 was accompanied by increased TPC and TFC levels, suggesting that enhanced enzymatic hydrolysis contributed to the release and transformation of phenolic compounds during fermentation. β-GC-mediated deglycosylation has been recognized as a major pathway for converting flavonoid glycosides into more bioactive aglycones, thereby improving the extractability and functionality of phenolic compounds [4]. Notably, LU4 simultaneously exhibited the highest β-GC activity and TFC, indicating that logarithmic-phase ultrasonication not only promoted enzyme production but also enhanced flavonoid biotransformation efficiency. Interestingly, although LU5 exhibited lower β-GC activity than LU4, it still maintained relatively high metabolic activity, as reflected by its sugar utilization and acidification capacity. This observation suggests that ultrasonication applied during different growth phases may regulate distinct metabolic processes. Logarithmic-phase ultrasonication primarily enhanced enzymatic biotransformation associated with phenolic metabolism, whereas stationary-phase ultrasonication may preferentially influence adaptive metabolic pathways. These growth phase-dependent responses further support the hypothesis that the physiological state of *L. acidophilus* determines the metabolic consequences of ultrasonic stimulation.

### 3.5 Effects of low-frequency ultrasonication on antioxidant activity

Numerous studies have demonstrated that phenolics and flavonoids exert important biological activities, including antioxidant effects [32], [33]. Therefore, DPPH and ABTS^+^ radical-scavenging assays were employed to evaluate the effects of low-frequency ultrasonication on the antioxidant capacity of fermented *C. aurantium* juice. As shown in Fig. 3D, fermentation significantly enhanced the DPPH radical-scavenging activity from 46.8 % (C) to 50.5 % (L), indicating improved antioxidant capacity after *L. acidophilus* fermentation. Ultrasonication further enhanced DPPH scavenging activity, with LU3, LU4, and LU5 exhibiting the highest values among all treatments (approximately 51.0–52.0 %). In contrast, ultrasonication applied during the lag phase (LU1 and LU2) or at high intensity during the stationary phase (LU6) produced only limited improvements. The enhanced antioxidant activity may be attributed to ultrasound-induced improvements in microbial metabolism and enzymatic biotransformation, which promoted the release of antioxidant compounds from the juice matrix, such as polyphenols and flavonoids. Similar results were reported by Chen et al. [13], who found that moderate ultrasound-assisted fermentation enhanced the antioxidant activity of ginkgo kernel juice. Previous studies have demonstrated that the DPPH radical-scavenging activity is strongly associated with the content, availability, and structural characteristics of phenolic compounds, particularly the number and position of phenolic hydroxyl groups, which determine their hydrogen-donating capacity [34]. In the present study, the variation pattern of DPPH scavenging activity was generally consistent with those observed for TPC, TFC, and β-GC activity, suggesting that ultrasonication promoted the release and transformation of antioxidant-active phenolics.

The superior antioxidant performance of LU4 corresponded well with its elevated β-glucosidase activity and TFC, indicating enhanced flavonoid biotransformation during logarithmic-phase ultrasonication. Notably, although LU5 exhibited lower flavonoid accumulation than LU4, its relatively high ABTS^+^ scavenging activity suggests that antioxidant enhancement may also involve other metabolite classes generated during fermentation.

ABTS^+^ radical-scavenging activity reflects the overall antioxidant capacity of both hydrophilic and lipophilic compounds. As illustrated in Fig. 3E, all fermented groups exhibited higher ABTS^+^ scavenging activity than the unfermented control (C), confirming the beneficial effect of *L. acidophilus* fermentation on antioxidant potential. Among all treatments, LU4 exhibited the highest ABTS^+^ radical-scavenging activity (61.1 %), followed by LU5 (58.7 %) and LU3 (56.8 %). These findings indicate that the beneficial effect of ultrasonication on antioxidant activity was dependent on both the growth stage of the microorganism and ultrasound intensity. During the logarithmic phase, mild ultrasonic cavitation may stimulate microbial metabolism and facilitate the release of intracellular enzymes, thereby accelerating the bioconversion of bound phenolics into antioxidant-active compounds. Consequently, LU3-LU5 exhibited stronger ABTS^+^ scavenging activity than the other treatments. Similar enhancements in antioxidant capacity following ultrasound-assisted fermentation have been reported in fruit- and plant-based fermentation systems, where ultrasonication promoted phenolic release and improved the ABTS^+^ radical-scavenging activity [30]. Moreover, the variation pattern of ABTS^+^ scavenging activity was generally consistent with those observed for TPC, TFC, and β-GC activity, suggesting that phenolic transformation and flavonoid metabolism contributed substantially to the improved antioxidant properties of the fermented juice.

Overall, these results indicate that ultrasonic intervention during the logarithmic and stationary growth phases is more effective than that during the lag phase in enhancing the antioxidant potential of fermented *C. aurantium* juice. These findings further demonstrate that the efficacy of ultrasonication is highly dependent on both microbial physiological state and ultrasonic intensity, emphasizing the importance of optimizing ultrasound parameters to maximize the functional quality of fermented products.

### 3.6 Metabolite profiling analysis

Based on the above results, LU4 and LU5 exhibited superior functional properties compared with the other ultrasonication groups. Therefore, the L, LU4 and LU5 groups were selected for integrated untargeted metabolomics to further elucidate the metabolic mechanisms underlying the differential effects of ultrasonic intervention at distinct growth phases. A total of 1,198 metabolites were tentatively identified and classified into 11 chemical superclasses (Fig. 4A). Among them, phenylpropanoids and polyketides constituted the largest category (24.37 %), followed by lipids and lipid-like molecules (19.01 %), organic acids and derivatives (18.09 %), and organic oxygen compounds (15.75 %). The predominance of phenylpropanoids and polyketides indicates that flavonoid-related metabolites are major constituents of *C. aurantium* juice and may contribute substantially to its antioxidant properties. Meanwhile, the high abundance of organic acids and carbohydrate-related metabolites reflects active microbial metabolism during fermentation. These metabolite classes may represent key biochemical targets through which ultrasonic treatment at different growth phases reshapes microbial metabolism and generates distinct metabolic signatures.

**Fig. 4:**
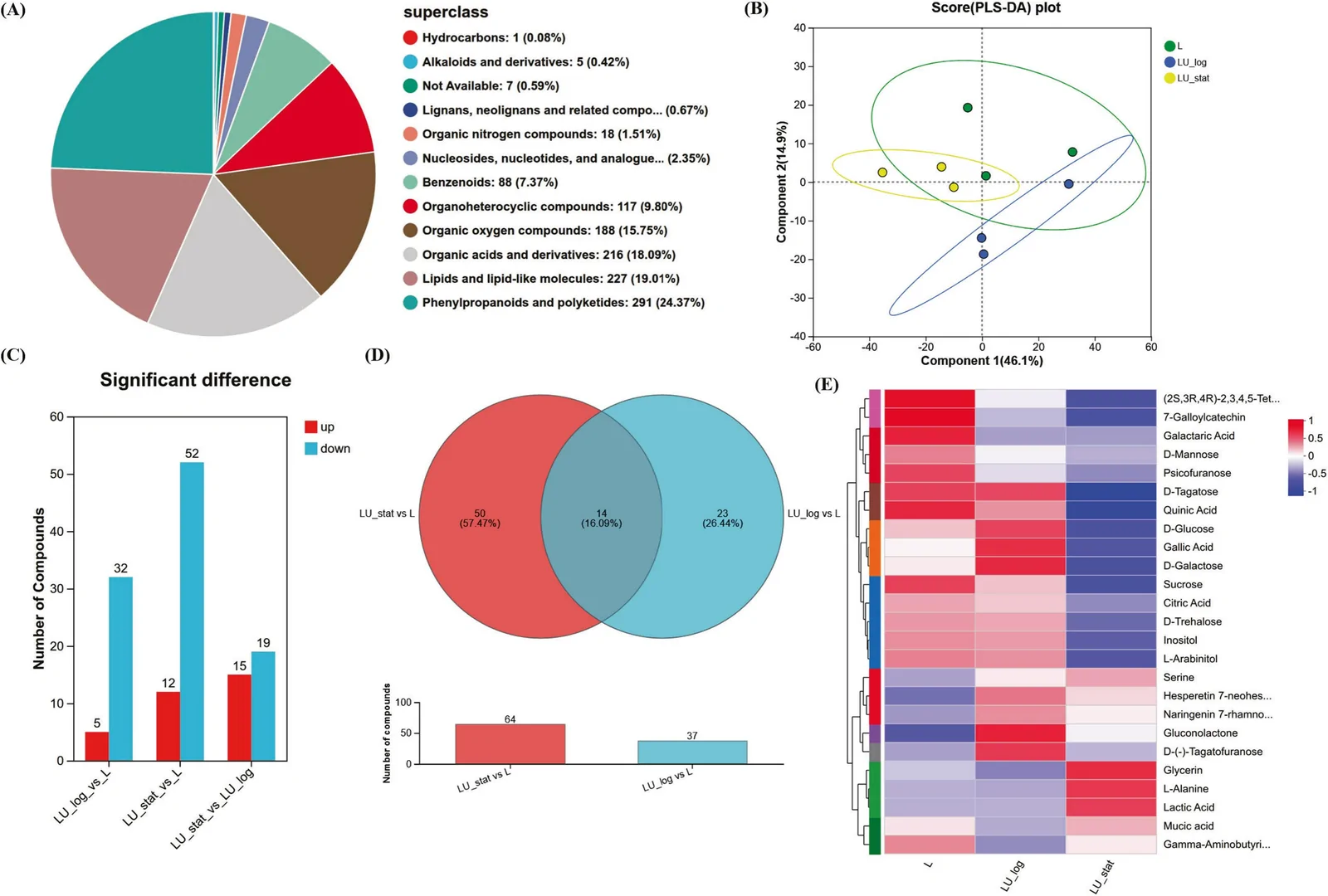
Metabolic profiling and differential metabolite analysis of *C. aurantium* L. juice fermented by *L. acidophilus* with ultrasonication applied at different growth phases. (A) Classification of identified metabolites based on GC–MS and LC-MS analyses. (B) PLS-DA score plot showing the metabolic separation among L, LU_log and LU_stat groups. (C) Numbers of differential metabolites in pairwise comparisons. (D) Venn diagram showing shared and unique differential metabolites among comparison groups. (E) Hierarchical clustering heatmap of representative differential metabolites. L: *C. aurantium* L. juice fermented by *Lactobacillus acidophilus* without ultrasonication. LU_log: Ultrasonication treatment applied during the logarithmic growth phase. LU_stat: Ultrasonication treatment applied during the stationary growth phase.

### 3.7 Analysis of differential metabolites

To further elucidate how ultrasonic treatment applied at different growth phases reshaped the metabolic landscape of fermented *C. aurantium* juice, differential metabolite (DM) screening was subsequently performed among the L, LU4, and LU5 groups. Partial least squares discriminant analysis (PLS-DA) showed that the L, LU4, and LU5 groups were clearly separated, indicating that ultrasonic intervention markedly altered the metabolic profile of *C. aurantium* juice compared with non-ultrasonicated juice (Fig. 4B). Compared with the non-ultrasonicated juice, 37 DMs were identified in LU4, including 5 up-regulated and 32 down-regulated metabolites, whereas 64 DMs were detected in LU5, including 12 up-regulated and 52 down-regulated metabolites (Fig. 4C). Moreover, LU5 and LU4 shared only 14 DMs relative to L, while 50 and 23 metabolites were specifically altered in LU5 and LU4, respectively (Fig. 4D), suggesting that ultrasonic treatment at different growth phases induced distinct metabolic remodeling patterns. The heatmap further showed that sugars and sugar derivatives, such as D-glucose, D-galactose, sucrose, D-tagatose, and D-trehalose, were generally decreased after ultrasound-assisted fermentation, whereas several fermentation-related metabolites, including lactic acid, L-alanine, glycerin, and γ-aminobutyric acid, were enriched, particularly in LU5 (Fig. 4E). These changes may be attributed to the enhancement of microbial membrane permeability, mass transfer efficiency, and intracellular enzymatic activities by low-frequency ultrasound, thereby promoting carbohydrate utilization and subsequent organic acid and amino acid metabolism [10], [35]. Previous studies have similarly reported that ultrasound accelerated substrate conversion and metabolic turnover [22]. Collectively, these findings indicate that LU4 and LU5 generated distinct metabolic signatures relative to the L group, reflecting growth phase-dependent metabolic responses to ultrasonic stimulation.

To further elucidate the growth phase-dependent metabolic responses to ultrasonic treatment, DMs between LU4 and LU5 were further compared. The orthogonal partial least squares discriminant analysis (OPLS-DA) score plot revealed a clear separation between the two groups, indicating that ultrasonic treatment applied during the logarithmic phase (119.17 W/L) and stationary phase (83.6 W/L) generated distinct metabolic signatures (Fig. 5A). According to the VIP ranking and heatmap analyses, LU4 was characterized by higher abundances of several carbohydrates and phenolic-related metabolites, including D-glucose, D-galactose, sucrose, D-tagatose, gallic acid, taxifolin, quinic acid, and 7-galloylcatechin (Fig. 5B and C). Among these metabolites, gallic acid, taxifolin, and 7-galloylcatechin possess strong antioxidant activities and have been widely reported as important contributors to the antioxidant capacity of plant-derived foods [36]. Their enrichment is therefore consistent with the higher TPC, TFC, DPPH, and ABTS^+^ values observed in LU4. Moreover, quinic acid is a key intermediate of the shikimate pathway and serves as a precursor for the biosynthesis of various phenolic compounds [37]. Together, these findings suggest that LU4 provided a metabolic environment more conducive to the accumulation of phenolic and antioxidant-related metabolites. In addition, the relatively higher levels of residual sugars in LU4 indicate that ultrasonic stimulation during the logarithmic phase may promote metabolite biosynthesis while preventing excessive substrate depletion. In contrast, LU5 exhibited significantly higher levels of fermentation-derived metabolites, including lactic acid, γ-aminobutyric acid (GABA), L-alanine, serine, glycerin, and mucic acid. Lactic acid is the major end-product of LAB fermentation and reflects enhanced carbohydrate catabolism, whereas GABA is a bioactive metabolite associated with antioxidant, neuroprotective, and stress-relieving functions [38]. The accumulation of L-alanine and serine further suggests active amino acid turnover and nitrogen metabolism during the stationary phase. These findings suggest that stationary-phase ultrasound may promote carbohydrate catabolism and amino acid metabolism while inducing adaptive stress responses, thereby facilitating the accumulation of fermentation-derived functional metabolites such as lactic acid and GABA. The enrichment of lactic acid, GABA, L-alanine, and serine in LU5 may be associated with the enhanced stress tolerance and adaptive metabolism of LAB cells in the stationary phase. Previous studies have demonstrated that stationary-phase LAB exhibit self-induced multistress resistance and activate metabolic pathways involved in energy conservation and stress adaptation [39], which may explain the preferential accumulation of fermentation-derived metabolites observed in LU5.

**Fig. 5:**
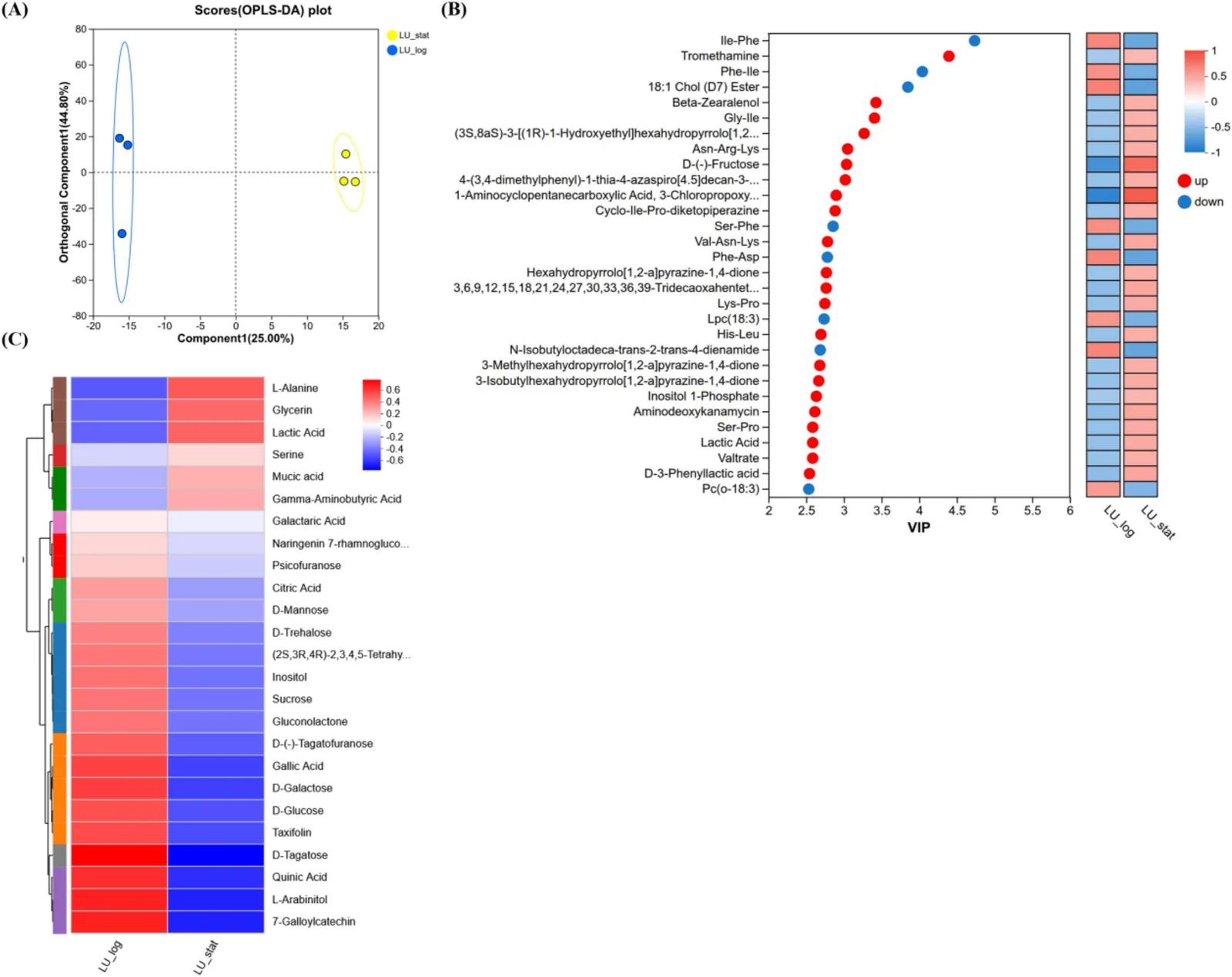
Growth phase-dependent metabolic alteration induced by low-frequency ultrasonication during *Lactobacillus acidophilus* fermentation of *Citrus aurantium* L. juice. (A) OPLS-DA score plot showing the metabolic separation between LU_log and LU_stat groups. (B) VIP plot displaying the key differential metabolites contributing to the discrimination between groups. (C) Hierarchical clustering heatmap of representative differential metabolites. LU_log: low-frequency ultrasonication (119.17 W/L) applied during the logarithmic growth phase. LU_stat: low-frequency ultrasonication (83.6 W/L) applied during the stationary growth phase.

Collectively, the results indicate that ultrasonic intervention at different growth phases redirected microbial metabolism toward distinct biochemical outcomes. LU4 preferentially accumulated flavonoid- and phenolic-related antioxidant metabolites, whereas LU5 favored the production of organic acids and amino acid-derived functional metabolites. These findings demonstrate that the physiological state of LAB at the time of ultrasonic exposure is a key determinant of metabolic remodeling during ultrasound-assisted fermentation.

### 3.8 KEGG enrichment analysis of differential metabolites in ultrasonication-treated juice

To gain further insight into the metabolic pathways affected by ultrasonic treatment, KEGG enrichment analysis was performed based on the DMs identified in LU4 and LU5 relative to the L group (Fig. 6). As shown in Fig. 6A, the pathways significantly enriched in LU4 mainly included glycine, serine and threonine metabolism, citrate cycle (TCA cycle), ascorbate and aldarate metabolism, arginine and proline metabolism, glutathione metabolism, flavonoid biosynthesis, flavone and flavonol biosynthesis, and phenylpropanoid biosynthesis. Notably, several pathways associated with phenolic and flavonoid metabolism were significantly enriched. The enrichment of these pathways was consistent with the higher levels of gallic acid, taxifolin, and 7-galloylcatechin observed in LU4. These pathways are closely associated with the biosynthesis of antioxidant compounds and may partially explain the enhanced TPC, TFC, DPPH, and ABTS^+^ values observed following logarithmic-phase ultrasonic treatment. Furthermore, the enrichment of the TCA cycle and amino acid metabolism pathways suggests that ultrasonic stimulation during the logarithmic phase enhanced metabolic activity and precursor supply, thereby facilitating the biosynthesis of secondary metabolites. In contrast, LU5 exhibited enrichment primarily in pathways related to amino acid metabolism and cellular maintenance, including alanine, aspartate and glutamate metabolism, arginine biosynthesis, nitrogen metabolism, D-amino acid metabolism, cysteine and methionine metabolism, and vitamin B6 metabolism. Furthermore, propanoate metabolism, purine metabolism, and nucleotide metabolism were also enriched, indicating active carbon utilization and cellular metabolic turnover. These results were consistent with the elevated levels of lactic acid, GABA, alanine, and serine observed in LU5, suggesting that stationary-phase ultrasonic treatment favored metabolic processes associated with amino acid conversion, nitrogen utilization, and adaptive metabolism. Previous studies have also reported that stationary-phase LAB exhibit enhanced stress resistance and activate pathways related to energy conservation and cellular adaptation [40], which may contribute to the preferential accumulation of fermentation-derived functional metabolites in LU5. Overall, the KEGG enrichment results further confirmed that ultrasonic intervention at different growth phases triggered distinct metabolic responses. Logarithmic-phase ultrasonic treatment preferentially promoted phenylpropanoid and flavonoid metabolism, whereas stationary-phase ultrasonic treatment mainly enhanced amino acid and nitrogen metabolism, ultimately leading to different metabolic remodeling patterns in fermented *C. aurantium* juice.

**Fig. 6:**
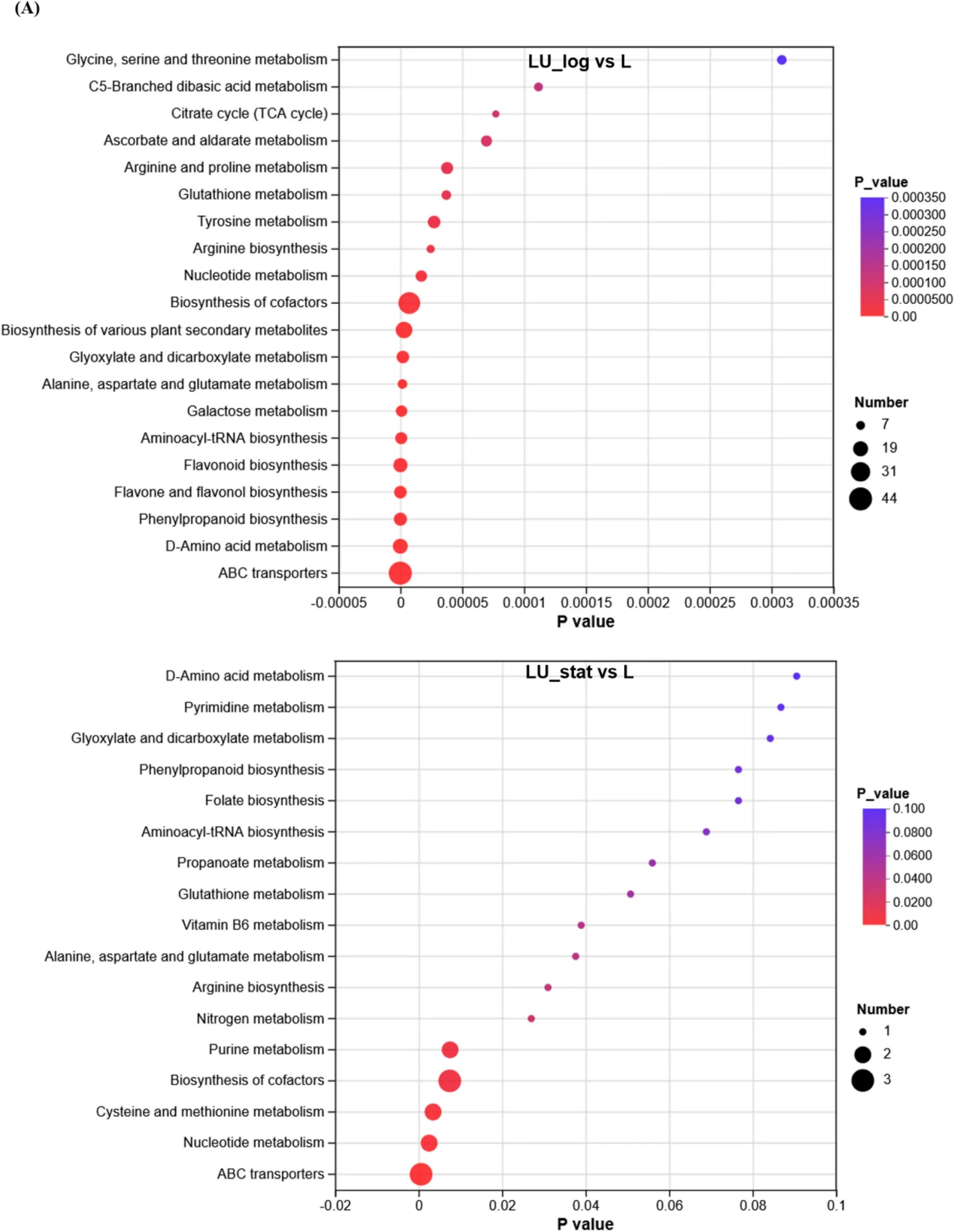
KEGG pathway enrichment analysis of differential metabolites in *L. acidophilus*-fermented *C. aurantium* juice subjected to low-frequency ultrasonication at different growth phases. (A) Enriched metabolic pathways of differential metabolites in the LU_log vs L comparison. (B) Enriched metabolic pathways of differential metabolites in the LU_stat vs L comparison. L: *C. aurantium* L. juice fermented by *Lactobacillus acidophilus* without ultrasonication. LU_log: low-frequency ultrasonication (119.17 W/L) applied during the logarithmic growth phase. LU_stat: low-frequency ultrasonication (83.6 W/L) applied during the stationary growth phase.

### 3.9 Correlation analysis between differential metabolites and antioxidant capacity

To further elucidate the relationships between functional properties and key DMs, Pearson correlation analysis was performed between antioxidant-related indicators and representative metabolites (Fig. 7). The results revealed that several phenolic and flavonoid-related metabolites, including taxifolin, 7-galloylcatechin, and gallic acid, were positively correlated with TPC, TFC, DPPH, and ABTS^+^ activities. Among them, taxifolin and 7-galloylcatechin exhibited particularly strong positive correlations with antioxidant indicators, suggesting that these compounds may contribute substantially to the enhanced antioxidant capacity of ultrasonication-treated juice. As important flavonoid and phenolic metabolites, these compounds possess strong free radical-scavenging activities and have been widely reported as major contributors to the antioxidant properties of plant-derived foods [41]. In addition, gallic acid showed significant positive correlations with taxifolin and 7-galloylcatechin, indicating coordinated regulation within phenylpropanoid and flavonoid biosynthetic pathways. Conversely, fermentation-derived metabolites such as lactic acid, GABA, L-alanine, and D-3-phenyllactic acid displayed positive correlations with β-GC activity and antioxidant indices. GABA, in particular, exhibited significant positive associations with ABTS^+^ and DPPH activities, suggesting that amino acid-derived metabolites also contributed to the functional improvement of fermented juice. The significant correlations between β-GC activity and several key metabolites further suggest that enzymatic biotransformation played an important role in shaping the metabolic profile of fermented juice. Enhanced β-GC activity may facilitate the conversion of glycosylated precursors into bioactive aglycones and their derivatives, thereby contributing to the accumulation of antioxidant-related metabolites and the subsequent improvement of functional properties [4]. These results provide additional evidence that metabolic remodeling is closely associated with the enhancement of juice quality during ultrasound-assisted fermentation.

**Fig. 7:**
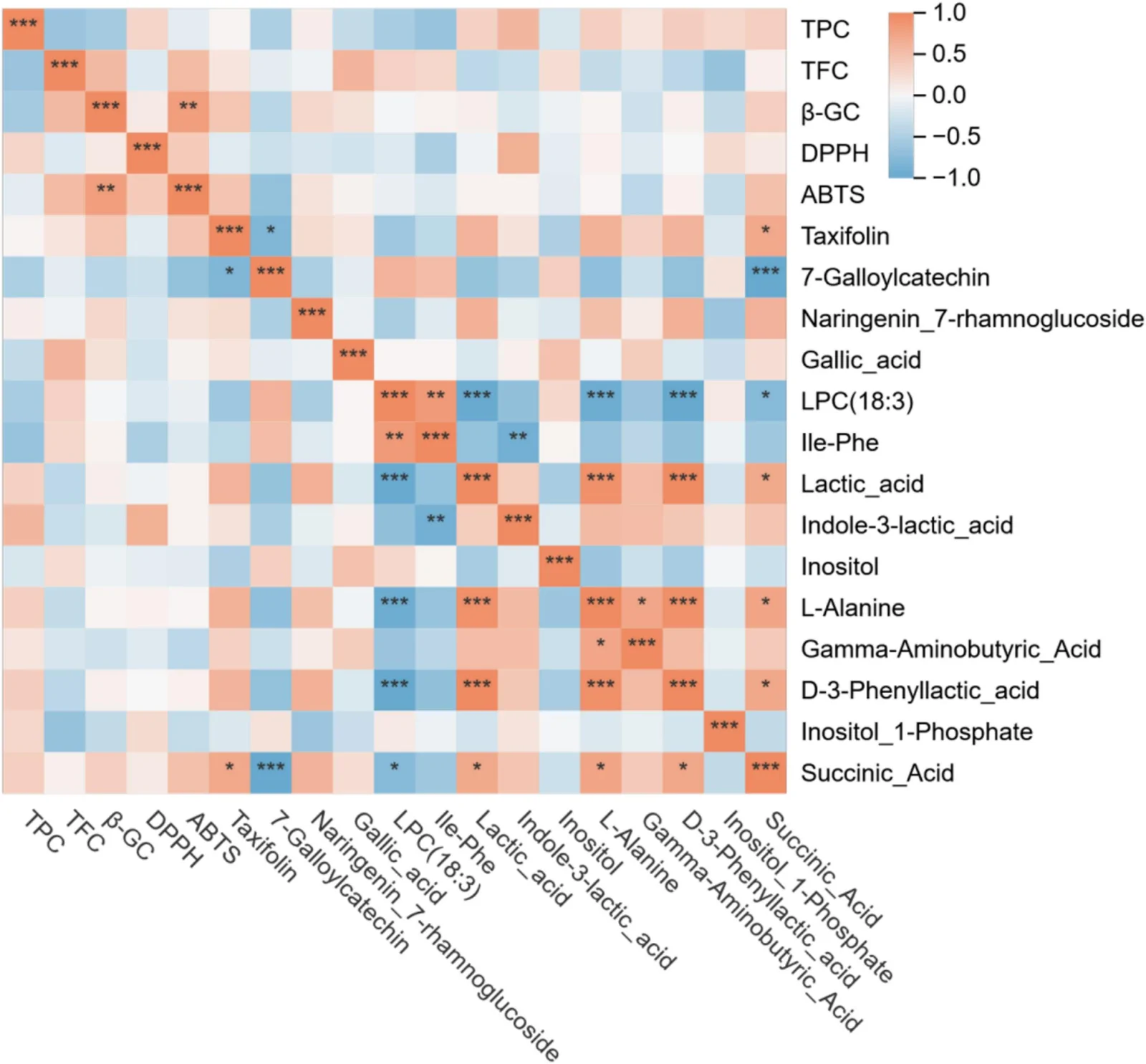
Correlation analysis between key differential metabolites and functional quality indicators in *L. acidophilus*-fermented *C. aurantium* juice. Red and blue colors indicate positive and negative correlations, respectively. *, **, and *** represent significant correlations at *P* < 0.05, *P* < 0.01, and *P* < 0.001, respectively.

From an industrial perspective, these findings suggest that growth phase-targeted low-frequency ultrasonication could be incorporated into juice fermentation to enhance microbial activity and functional metabolite formation through cavitation-induced microstreaming, increased membrane permeability, and improved mass transfer. Applying a brief treatment only at the most responsive growth stage may also avoid unnecessary energy input compared with continuous sonication. This approach therefore shows potential for developing fermented functional juices with improved bioactive profiles. Nevertheless, its environmental and economic advantages require pilot-scale verification, considering electricity and water consumption, equipment investment and maintenance, acoustic-field uniformity, and processing throughput. Future studies should determine energy consumption and production cost per liter and perform life-cycle and techno-economic assessments. Furthermore, because only *L. acidophilus* LA137 was evaluated, the observed growth phase-dependent responses to ultrasonication should be considered strain-specific. Further studies involving multiple *L. acidophilus* strains are required to determine whether these responses can be generalized at the species level.

## 4 Conclusions

This study demonstrated that low-frequency ultrasonication effectively enhanced *L. acidophilus* LA137 fermentation of *C. aurantium* juice in a growth phase-dependent manner. Compared with non-sonicated fermentation and lag-phase ultrasonication, treatments applied during the logarithmic and stationary phases showed greater improvements in microbial growth, sugar utilization, β-GC activity, phenolic/flavonoid accumulation, and antioxidant capacity, with LU4 and LU5 exhibiting the best overall performance. Integrated untargeted metabolomics revealed distinct metabolic profiles between LU4 and LU5. LU4 was mainly associated with phenylpropanoid and flavonoid metabolism and showed increased levels of gallic acid, taxifolin, and 7-galloylcatechin, whereas LU5 was associated with organic acid and amino acid metabolism and showed higher levels of lactic acid, GABA, L-alanine, and serine (Fig. 8). Although comparisons between L and LU1-LU6 revealed the overall effects of ultrasonication within the inoculated fermentation system, the absence of an uninoculated sonicated control prevented complete separation of the direct effects of ultrasound on juice components from bacteria-mediated effects. This limitation should be addressed in future studies. Overall, these findings provide new insights into the growth phase-dependent metabolic patterns of ultrasound-assisted *L. acidophilus* fermentation and provide a basis for optimizing ultrasonication in the development of functional fermented fruit juices.

**Fig. 8:**
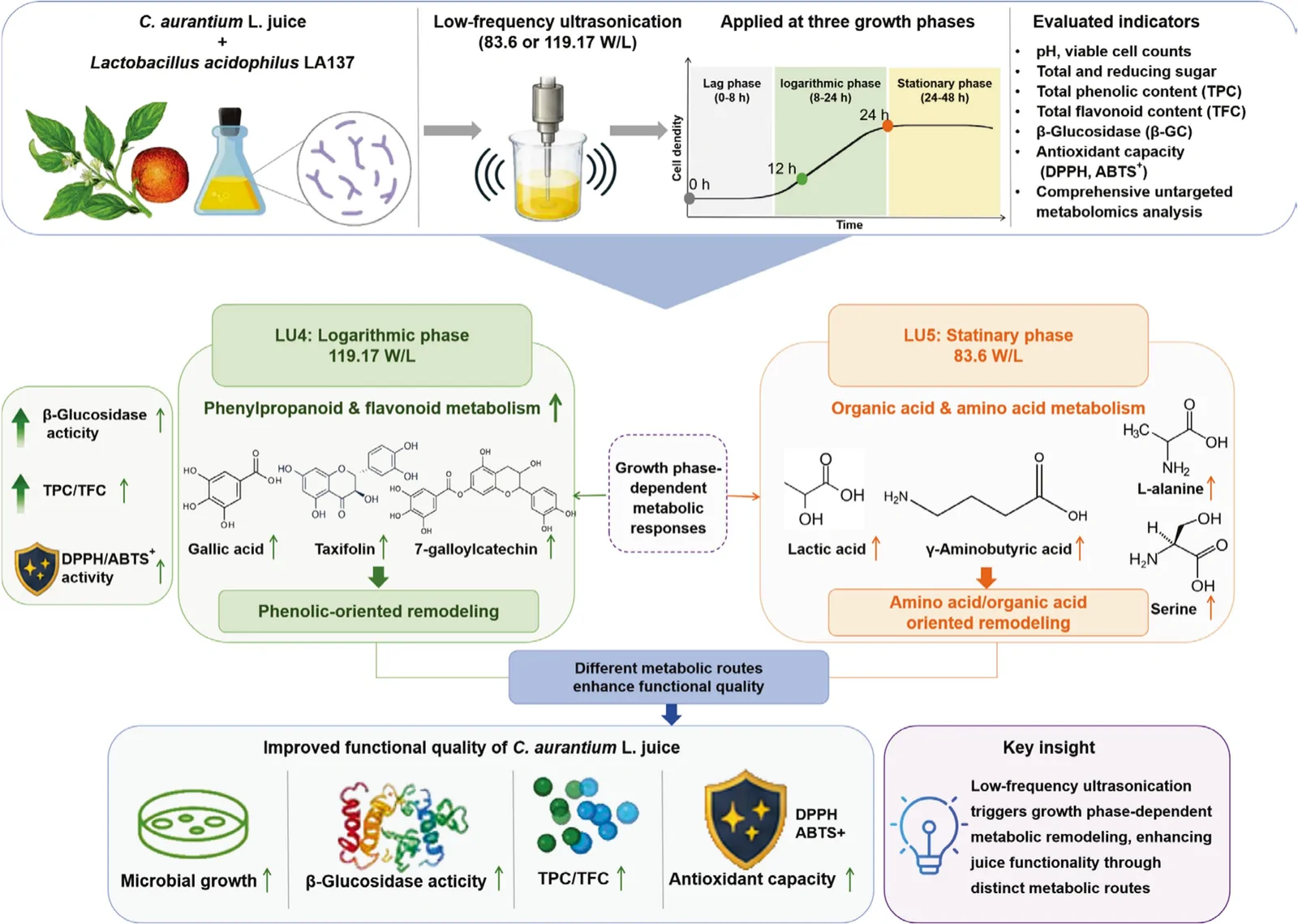
Proposed mechanism of growth phase-dependent metabolic reprogramming induced by low-frequency ultrasonication, leading to improved functional quality of *Lactobacillus acidophilus*-fermented *Citrus aurantium* L. juice. LU4: low-frequency ultrasonication (119.17 W/L) applied during the logarithmic growth phase. LU5: low-frequency ultrasonication (83.6 W/L) applied during the stationary growth phase.

## Data availability

Data will be made available on request.

## CRediT authorship contribution statement

**Binbin Li:** Writing – review & editing, Writing – original draft, Methodology, Investigation, Formal analysis. **Ningning Shen:** Validation, Software, Investigation, Data curation. **Linxiao Wang:** Writing – review & editing, Methodology, Data curation, Conceptualization. **Shun Li:** Writing – review & editing, Visualization, Validation, Investigation. **Longquan Xiao:** Software, Formal analysis, Conceptualization. **Lin Zhou:** Writing – review & editing, Visualization, Resources. **Zhengnan Ren:** Writing – review & editing, Project administration, Methodology, Investigation. **Xinhui Wang:** Writing – review & editing, Supervision, Project administration, Funding acquisition.

## Declaration of competing interest

The authors declare that they have no known competing financial interests or personal relationships that could have appeared to influence the work reported in this paper.
